# A systematic review of outcomes of wrist arthrodesis and wrist arthroplasty in patients with rheumatoid arthritis

**DOI:** 10.1177/1753193420953683

**Published:** 2020-09-17

**Authors:** Xi Ming Zhu, Edward Perera, Chetan Gohal, Brittany Dennis, Moin Khan, Bashar Alolabi

**Affiliations:** 1 St. George’s University Hospitals NHS Foundation Trust, University of London, London, UK; 2 Division of Orthopaedic Surgery, McMaster University, Hamilton, Ontario, Canada

**Keywords:** Arthrodesis, arthroplasty, wrist, arthritis, rheumatoid arthritis, wrist fusion, wrist replacement

## Abstract

Surgical management of end-stage rheumatoid wrists is a contentious topic. The standard surgical treatment has traditionally been wrist arthrodesis. Wrist arthroplasty, however, offers an alternative that preserves some wrist motion. A systematic review of MEDLINE, EMBASE and CENTRAL databases was conducted. Data from 23 studies representing 343 cases of wrist arthrodesis and 618 cases of wrist arthroplasty were included. Complication rates were 17% for arthrodesis and 19% for arthroplasty, and both procedures were effective at alleviating pain and improving grip strength. Functional assessment by Disabilities of the Arm, Shoulder, and Hand and Patient-Related Wrist Evaluation of arthroplasty patients revealed clinically meaningful functional improvement compared with preoperative measurements. In contrast to previously published findings both procedures demonstrated comparable complication rates. While this can be speculated to be from advancements in prosthetics, robust long-term follow-up data on wrist arthroplasty are not available yet.

## Introduction

Prognosis for rheumatoid arthritis (RA) has improved drastically with the advent of disease-modifying antirheumatic drugs in the past few decades (Pisetsky, 2003). Surgical management of painful, end-stage arthritis of the wrist, however, is still often indicated.

Wrist arthrodesis has long been the surgical modality of choice for degenerative or inflammatory arthritis, providing stability and effectively alleviating pain (Hayden and Jebson, 2005). Precontoured plates and screws that allow rigid fixation and compression are often used (Wei and Feldon, 2017). Arthrodesis can be performed in conjunction with a proximal row carpectomy. Despite its effectiveness in providing pain relief, wrist arthrodesis eliminates all motion (Hayden and Jebson, 2005).

Wrist arthroplasty is indicated in low-demand patients interested in a motion-preserving option that will facilitate performance of activities of daily living (Weiss et al., 2013). The prosthesis offers a balance of wrist strength and stability while preserving some range of motion (ROM) and diminishing pain (Adams, 2004; Meuli, 1980; Swanson, 1973). Implant designs have changed tremendously over the years. Swanson introduced silicone implants in the 1970s (Adams, 2004; Swanson, 1973), but long-term studies showed mechanical failure and inflammatory reactions to the silicone (Brase and Millender, 1986; Jolly et al., 1992). Transition was made to ball-and-socket oriented metal implants followed by metal-on-plastic hemispheric designs in the 1990s (Meuli, 1984; Volz, 2006). Issues with instability, loosening and dislocation resulted in many recalls of these products. The latest ‘anatomical’ wrist arthroplasty design addresses these issues by treating the wrist as a two-bone joint through distal radius and carpal fixation and intercarpal arthrodesis (Adams, 2004; Cooney et al., 1984; Krukhaug et al., 2011; Nydick et al., 2013; Pfanner et al., 2017; Sagerfors et al., 2015).

Since a systematic review published by Cavaliere and Chung (2008), techniques have improved for wrist arthroplasty as demonstrated by a more recent review from Berber et al. (2018). This systematic review aims to provide an up-to-date comparison of wrist arthrodesis versus arthroplasty for patients with RA.

## Methods

This study was conducted according to the methodology described in the Cochrane Handbook for Systematic Reviews of Interventions (Higgins Julian and Green, 2011a) and is reported according to the Preferred Reporting Items for Systematic Reviews and Meta-Analyses (PRISMA) statement (Higgins et al., 2011).

### Search strategy and study screening

A comprehensive search of Ovid Medline, Ovid EMBASE and Cochrane Central Register of Controlled Trials (CENTRAL) was performed covering from 1 January 1946 until 23 September 2018. The search strategy can be found in Online Table S1. A manual search of the references of all eligible articles was performed to identify any relevant articles missed in the original search. The title, abstract and full-text screening was performed by two reviewers (Ming Zhu and Edward Perera) independently and in duplicate using piloted screening forms. Disagreements during title and abstract screening moved onto the next stage for more in-depth review. Any disagreements were discussed between reviewers, and a senior author (Chetan Gohal) was consulted for any remaining discrepancies.

### Inclusion and exclusion criteria

Included studies reported clinical and functional outcomes for RA patients receiving either wrist arthrodesis or arthroplasty with a minimum of 12 months follow-up. No restrictions were placed on disease classification, but all patients reported had failed non-surgical management. Exclusion criteria included any forms of arthritis other than rheumatoid (osteoarthritis, psoriatic and postinfectious), non-surgical management, non-English studies, non-human studies, revision procedures and other procedures (proximal row carpectomy, partial arthrodesis and interpositional arthroplasty). In studies that used the same population, the study with the larger patient pool was used. Case reports, editorials, reviews, expert opinions and basic science papers were excluded.

### Assessment of methodological quality

Methodological quality of the included studies was assessed in duplicate (by Ming Zhu and Edward Perera) using the Methodological Index for Non-Randomized Studies (MINORS) instrument (Slim et al., 2003) for non-randomized studies and the Cochrane risk of bias tool for randomized controlled trials (Higgins et al., 2015).

### Data abstraction

All data were abstracted in duplicate using piloted screening forms with disagreement resolution resolved through discussion. Information extracted included author, date of publication, journal of publication, number of participants, description of study population (e.g. age, important comorbidities), study eligibility criteria, description of surgical procedure, prosthetic of choice, indication for surgery, measurement of pre- and postoperative wrist function, ROM, grip strength, pain severity and adverse events or complications. Postoperative outcomes scores such as Patient-Rated Wrist Evaluation (PRWE) and Disabilities of the Arm, Shoulder, and Hand (DASH) were also abstracted.

### Statistical analysis

Results are presented descriptively, and statistical pooling was performed when possible. Agreement levels between the independent data extractors were assessed using Cohen’s kappa statistic. On the basis of the guidelines of Landis and Koch, a kappa of 0 to 0.2 represents slight agreement, 0.21 to 0.40 fair agreement, 0.41 to 0.60 moderate agreement and 0.61 to 0.80 substantial agreement (Landis and Koch, 1977). A value above 0.80 is considered almost perfect agreement.

### Literature search

The literature search and screening results are shown in Online Figure S1. Agreement on study inclusion between the reviewers for title was fair (k: 0.380; SE: 0.049), at the abstract stage was moderate (k: 0.444; SE: 0.071) and full text was moderate (k: 0.473; SE: 0.099).

Of the six studies examining patients who underwent arthrodesis, one was prospective (Masada et al., 2003) while the remainder were retrospective (Onuma et al., 2015; Rauhaniemi et al., 2005; Rehak et al., 2000; Toma et al., 2007; Vahvanen and Tallroth, 1984). Of the 15 studies evaluating arthroplasty patients, seven studies were prospective (Cobb and Beckenbaugh, 1996; Divelbiss et al., 2002; Ferreres et al., 2011; Figgie et al., 1988; Harlingen et al., 2011; Herzberg et al., 2012; Ward et al., 2011), and eight studies were retrospective (Kistler et al., 2005; Lirette and Kinnard, 1995; Figgie et al., 1990; Pfanner et al., 2017; Radmer et al., 2003, 1999; Summers and Hubbard, 1984; Takwale et al., 2002). Two studies compared arthrodesis versus arthroplasty (Murphy et al., 2003; Vicar and Burton, 1986). Patient demographics are shown in Table 1.

**Table 1.** Patient demographics for arthrodesis and arthroplasty groups.

	Arthrodesis	Arthroplasty
Number of studies featuring each procedure	8	17
Number of patients	314	560
Total number of primary operations	343	618
Mean age (years)	53.4	57.1
Mean follow-up period (years)	3.9	5.5

Study characteristics and MINORs scores can be found in Online Table S2. The mean MINORs score was 10.8 (range 8–14) for the six arthrodesis studies. The mean MINORs score for the 15 arthroplasty studies was 12.8 (range 8–16). The mean MINORs score for the two comparative studies was 19.5.

## Results

### Functional outcomes

There was significant heterogeneity in the reporting of functional outcomes between studies, and non-validated assessment tools were frequently encountered. No arthrodesis study included in this review measured functional outcomes using validated assessment tools. Many arthroplasty studies that used DASH or PRWE lacked preoperative outcome scores (Table 2). The three studies with a combined 63 patients (Divelbiss et al., 2002; Harlingen et al., 2011; Ward et al., 2011) measuring both preoperative and postoperative DASH scores all indicated a functional improvement, with an improvement in scores ranging from 14 to 32. This improvement is clinically meaningful since the minimally clinical important difference for the DASH is 11 (Franchignoni et al., 2014). The objective scores received in two studies (Ferreres et al., 2011; Pfanner et al., 2017) with a total of 36 patients measuring PRWE had a mean postoperative score of 36.

**Table 2.** DASH (Disabilities of the Arm, Shoulder, and Hand) and PRWE (Patient-Rated Wrist Evaluation) scores both pre- and postoperatively for studies that reported them.

Study	Preoperative/ postoperative DASH score	Preoperative/ Postoperative PRWE score
Divelbiss et al., 2002	46, 32.1	NS, NS
Ferreres et al., 2011	NS, NS	NS, 29.6
Harlingen et al., 2011	66, 34	NS, NS
Herzberg et al., 2012	NS, 20	NS, NS
Pfanner et al., 2017	NS, 49	NS, 41.7
Ward et al., 2011	62, 40	NS, NS

NS: not stated.

### Pain

A summary of the pain findings for the studies can be found in Online Table S3. A meta-analysis could not be performed due to the inconsistent nature of pain measurement and reporting. Both arthrodesis and arthroplasty were effective in reducing pain. Of 314 arthrodesis patients, one was still in severe pain after surgery. Similarly, two patients of 560 who underwent arthroplasty were in severe pain.

### ROM

In studies that reported the outcome, there was loss of ROM across the flexion/extension and radial/ulnar deviation arcs for the arthrodesis group of 81 patients in two studies (Rehak et al., 2000; Vicar and Burton, 1986). Pronation/supination motion was maintained at a 145° arc postoperatively for the one study that reported this (Vicar and Burton, 1986). For the 538 patients receiving arthroplasty, ROM improved in all planes. The mean preoperative ROMs were 47° (35°–66°) for flexion/extension, 18° (9°–21°) for radial/ulnar deviation and 126° (104°–138°) for pronation/supination. The mean postoperative ROMs were 58° (38°–72°) for flexion/extension, 30° (20°–98°) for radial/ulnar deviation and 157° (141°–172°) for pronation/supination. More detailed results can be found in Online Table S4.

### Grip strength

It is difficult to objectively quantify grip strength improvement, largely due to inconsistent pre- and postoperative measurements, along with varying means of measurement (Table 3). Therefore, we interpreted grip strength as a percentage of the preoperative level. Change in grip strength postoperatively compared with preoperative assessment ranged from +22% to +130%, with an average increase in grip strength of 76% for arthrodesis (three studies, 177 patients), while the range was –66% to 76% with an average increase of 31% for arthroplasty (seven studies, 330 patients). Hence, patients can largely expect an increase in grip strength, regardless of the treatment method.

**Table 3.** Grip strength assessment pre- and postoperatively as reported in each study.

Study	Surgical intervention	Preoperative grip strength	Postoperative grip strength
Rauhaniemi et al., 2005	Arthrodesis	NS	24% when right wrist fused 20% when left wrist fused
Vahvanen and Tallroth, 1984	Arthrodesis	NS	2.2 kg
Cobb and Beckenbaugh, 1996	Arthroplasty	4.1 kg	5.9 kg
Harlingen et al., 2011	Arthroplasty	NS	13 kg
Herzberg et al., 2012	Arthroplasty	NS	40% stronger
Pfanner et al., 2017	Arthroplasty	NS	11 kg
Radmer et al., 2003	Arthroplasty	0.32 kgf/cm^2^	0.11 kgf/cm^2^
Radmer et al., 1999	Arthroplasty	18 kPa	29 kPa
Vicar and Burton, 1986	Arthrodesis	2.7 kg	6.2 kg
	Arthroplasty	2.9 kg	5.1 kg

NS: not stated.

### Patient satisfaction

In four studies that included 213 patients who had an arthrodesis, 65% of patients were satisfied with the operation (Onuma et al., 2015; Rauhaniemi et al., 2005; Rehak et al., 2000; Toma et al., 2007). The low satisfaction incidence was mainly influenced by the results of Rauhaniemi et al. (2005), who reported 42% of 115 patients were satisfied with their operation (Online Table S5). In patients receiving arthroplasty (319 patients in nine studies), 84% of patients were satisfied with outcome (Cobb and Beckenbaugh, 1996; Ferreres et al., 2011; Figgie et al., 1990; Harlingen et al., 2011; Herzberg et al., 2012; Kistler et al., 2005; Lirette and Kinnard, 1995; Radmer et al., 1999; Summers and Hubbard, 1984).

### Complications

The overall incidence of complications for arthrodesis was 17% (59 cases of 343 indexed operations) among eight studies reporting complications (Masada et al., 2003; Murphy et al., 2003; Onuma et al., 2015; Rauhaniemi et al., 2005; Rehak et al., 2000; Toma et al., 2007; Vahvanen and Tallroth, 1984; Vicar and Burton, 1986). The most common complication was carpal tunnel syndrome and prosthetic loosening, which each accounted for 17% of all complications. Other complications in this group included tendon adhesions (12%), extensor tendon irritation (7%), wound dehiscence (5%) and infections (3%).

The overall incidence of complications in arthroplasty was 19% (111 cases of 600 indexed operations) from 16 studies (Cobb and Beckenbaugh, 1996; Divelbiss et al., 2002; Ferreres et al., 2011; Figgie et al., 1988, 1990; Harlingen et al., 2011; Herzberg et al., 2012; Lirette and Kinnard, 1995; Murphy et al., 2003; Pfanner et al., 2017; Radmer et al., 1999, 2003; Summers and Hubbard, 1984; Takwale et al., 2002; Vicar and Burton, 1986; Ward et al., 2011). The most common complications were implant dislocation and loosening, accounting for 20% and 14%, respectively, of all complications. Other complications included wound infection (6%), limited ROM (7%), carpal tunnel syndrome (5%), stiffness (4%) and impingement (2%).

A subdivision of patients based on generation of prosthesis is as follows: 1st generation (three studies, 54 patients, 67 cases), 2nd generation (zero studies), 3rd generation (11 studies, 341 patients, 385 cases) and 4th generation (three studies, 165 patients, 166 cases). The complication incidence for arthroplasty was 18% for 1st generation, 22% for 3rd and 11% for 4th-generation components. A summary of surgical complications can be found in Online Table S6.

### Comparative studies

The two comparative studies (Murphy et al., 2003; Vicar and Burton, 1986) included 47 patients (60 cases) for arthrodesis and 50 patients (61 cases) for arthroplasty. Murphy et al. reported similar complication incidences for arthrodesis and arthroplasty at 13% and 14%, respectively. With regards to function, Murphy et al. (2003) reported little difference between the two treatment arms through DASH, PRWE and their study-specific surveys examining function. Vicar and Burton (1986) reported a complication incidence of 18% for arthrodesis (Millender/Nalebuff technique) and 25% for arthroplasty (Swanson 1st-generation prosthesis). In addition, they noted that 97% of their arthrodesis patients and 78% of their arthroplasty patients had a ‘good’ or ‘excellent’ result.

### Sub-analysis of 3rd- and 4th-generation arthroplasty prosthetics

A subanalysis, looking exclusively at the results of studies in which arthroplasty was performed with a 3rd- or 4th-generation prosthetic yielded similar results. Grip strength postoperatively ranged from –69% to +60% of preoperative grip strength with a mean improvement of +19%. Mean patient satisfaction was 93%, while the incidence of complications was 17%, lower than both the arthrodesis group and arthroplasty group as a whole.

## Discussion

Both surgical treatments exhibited similar complication incidences, arthrodesis at 17% and arthroplasty at 19%. This is contrary to historical findings of a higher complication incidence for wrist arthroplasty. Arthroplasty complications were primarily caused by prosthetic dislocation and loosening, while complications in arthrodesis patients were primarily attributable to carpal tunnel syndrome and tendon adhesions. The comparable results between surgical procedures contrast with previous findings by Cavaliere and Chung (2008), who reported complication incidences of 30% for arthroplasty and 17% for arthrodesis. This difference is likely attributable to advancements in prosthetic design, which have led to better clinical outcomes and survivorship (Kennedy and Huang, 2016).

The previous review included 2nd- and 3rd-generation prostheses exclusively (Cavaliere and Chung, 2008). The inclusion of newer 4th-generation prostheses in this review likely had an impact, as complications with this generation were lower than previous generations (11%). A recent systematic review of wrist arthroplasty by Berber et al. (2018) showed complication incidences ranging from 0.1% to 2.9%. The discrepancy between our findings and those of Berber et al. (2018) is that their review considered arthroplasties for all aetiologies, including osteoarthritis, whereas isolating rheumatoid patients in this study produces a higher complication incidence, as the natural pathogenesis of rheumatism precludes definitive disease curation with arthroplasty.

ROM is restricted due to arthrodesis, however, this does not necessarily correlate with satisfaction or function. A retrospective review by Wagner et al. (2015) found that patients who had received bilateral wrist arthrodesis were generally happy with their function and had adapted well, and 93% would repeat the surgery.

Grip strength for arthrodesis increased by a mean of 76% compared with preoperative assessments, while the increase was 31% for arthroplasty. Only two studies for arthrodesis provided sufficient results for grip strength analysis. The smaller increase in grip strength for arthroplasty may be attributable to Radmer’s study, which reported a decrease of 66% in grip strength postoperatively (Radmer et al., 2003), though no reasons were found. The mean value of grip strength improvement for arthrodesis of 76% matched the maximum value observed for the arthroplasty group, suggesting that arthrodesis may offer greater return of grip strength.

The two comparative studies (Murphy et al., 2003; Vicar and Burton, 1986) revealed comparable outcomes. Reasons provided for the lack of differentiation in function were thought to be due to a lack of functional deficit from arthrodesis, the motion offered by arthroplasty not having a noticeable improvement in function and insufficient sensitivity of DASH, PRWE and study-specific surveys in measuring wrist function (Murphy et al., 2003). Thus, arthrodesis and arthroplasty of the wrist are both effective in the management of refractory RA and are comparable in their clinical outcomes. Although arthroplasty offers greater preservation of wrist motion, there does not appear to be an obvious differentiation in function, pain relief or complications. A salient quantitative summary of pain improvement remains elusive due to the variation in pain assessment and reporting.

Limitations of this review include poor data reporting from the studies and heterogeneity in modalities of outcome reporting (i.e. differing methods of pain assessment). In addition, the 12-month minimum follow-up period may overlook long-term effects of both arthrodesis and arthroplasty. The practicality of extending the minimum follow-up period in our exclusion criteria was limited by the amount of remaining eligible studies for data extraction. A possible confounding factor is the improvement in the medical management of RA over the past decades. The advent of novel biological medications and deeper understanding of disease pathogenesis have resulted in both improved clinical outcomes and disease remission (Aletaha and Smolen, 2018). This is unlikely to have had an impact on the findings of this review as there is no indication that arthrodesis patients were not privy to the same medical management of their arthroplasty compatriots.

## Supplemental Material

sj-pdf-1-jhs-10.1177_1753193420953683 - Supplemental material for A systematic review of outcomes of wrist arthrodesis and wrist arthroplasty in patients with rheumatoid arthritis Click here for additional data file. Supplemental material, sj-pdf-1-jhs-10.1177_1753193420953683 for A systematic review of outcomes of wrist arthrodesis and wrist arthroplasty in patients with rheumatoid arthritis by Xi Ming Zhu, Edward Perera, Chetan Gohal, Brittany Dennis, Moin Khan and Bashar Alolabi in Journal of Hand Surgery (European Volume)
