# Supplementary material for: A systematic review of outcomes of wrist arthrodesis and wrist arthroplasty in patients with rheumatoid arthritis
Source: J Hand Surg Eur Vol. 2020 Sep 17;46(3):297–303. doi: 10.1177/1753193420953683 (PMC7897788; doi:10.1177/1753193420953683)
Supplement: sj-pdf-1-jhs-10.1177_1753193420953683 - Supplemental material for A systematic review of outcomes of wrist arthrodesis and wrist arthroplasty in patients with rheumatoid arthritis [file sj-pdf-1-jhs-10.1177_1753193420953683.pdf]

SUPPLEMENTAL MATERIAL

Online Figure S1. A summary of literature search and screening results in accordance with PRISMA guidelines.

Online Table S1 – Search strategies used for MEDLINE, EMBASE, and CENTRAL to generate a list of eligible articles for the review.

Online Table S2 – Summary of general characteristics for each of the studies included, including prosthetic generation, number of cases, number of wrists operated on, and mean follow-up in years.

Online Table S3 – Descriptions of pain scales used by each study and pain assessment summary pre and postoperatively.

Online Table S4 – Summary of the range of motion for each study across flexion/extension, radial/ulnar deviation, and pronation/supination. The values are tallied across each modality of movement (i.e. 30° of flexion and 10° of extension is reported as 40°).

Online Table S5 – Summary of patient satisfaction reported for arthrodesis and arthroplasty.

Online Table S6 – Total number of complications along with a breakdown of each specified type of complication for each study.

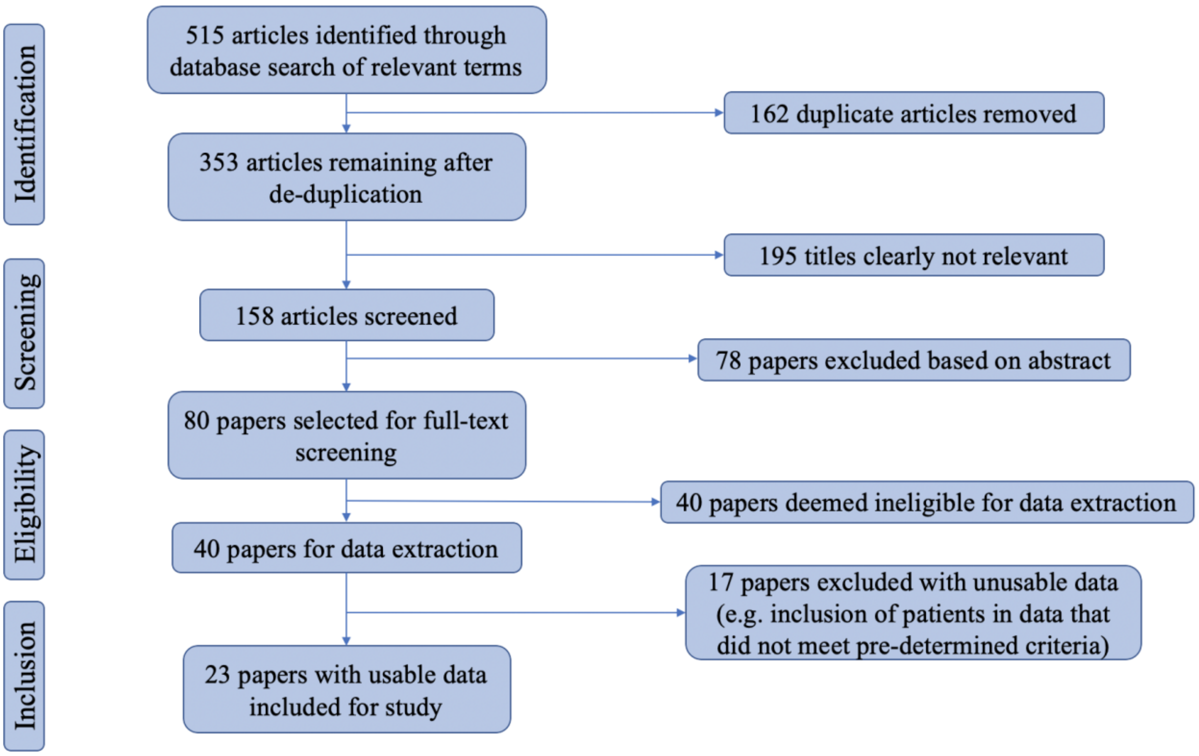

Online Figure S1. A summary of literature search and screening results in accordance with PRISMA guidelines.

Online Table S1. The search strategy used to identify applicable studies in MEDLINE, EMBASE, and CENTRAL.

MEDLINE	EMBASE	Cochrane Library Database
Strategy	Strategy	Strategy
1. Osteoarthritis	1. Osteoarthritis	1. Rheumatoid arthritis
2. Rheumatoid arthritis	2. Rheumatoid arthritis	2. Wrist arthrodesis
3. Wrist arthroplasty	3. Wrist arthroplasty	3. Wrist arthroplasty
4. Wrist arthrodesis	4. Wrist arthrodesis	4. Total wrist replacement
5. Total wrist replacement	5. Total wrist replacement	5. Total wrist arthroplasty
6. Total wrist arthroplasty	6. Total wrist arthroplasty	6. Wrist fusion
7. Wrist fusion	7. Wrist fusion	7. 2 or 3 or 4 or 5 or 6
8. 1 or 2	8. 1 or 2	8. 1 and 7
9. 3 or 4 or 5 or 6 or 7	9. 3 or 4 or 5 or 6 or 7	
10. 8 and 9	10. 8 and 9	
11. Limit 10 to English text only	11. Limit 10 to English text only	
12. Limit 11 to humans only	12. Limit 11 to humans only	

Online Table S2. Summary of article characteristics included for study.

Author (surname), Year	Study Design	Prosthesis (Generation) or technique used	Number of patients (number of wrists)	Intervention	Mean Age (years)	Follow-up (years)	MINORS Score
Cobb et al 1996	Prospective Cohort	Biaxial (3)	52 (64)	Arthroplasty	57.2	6	16
Divelbiss et al 2002	Prospective Cohort	Universal (3)	19 (22)	Arthroplasty	48.1	1.64	16
Ferreres et al 2011	Prospective Cohort	Universal 2 (4)	14 (14)	Arthroplasty	54	5.62	13
Figgie, H.E. et al 1988	Prospective Cohort	Trispherical (3)	32 (38)	Arthroplasty	60	5	8
Figgie, M. et al 1990	Retrospective Cohort	Trispherical (3)	26 (35)	Arthroplasty	58	9	14
Harlingen et al 2014	Prospective Cohort	Biaxial (3)	29 (32)	Arthroplasty	54	6	13
Herzberg et al 2012	Prospective Cohort	ReMotion (4)	129 (129)	Arthroplasty	63	4	16
Kistler et al 2005	Retrospective Cohort	Swanson silicone (1)	16 (18)	Arthroplasty	65.9	15.2	10
Lirette et al 1995	Retrospective Cohort	Biaxial (3)	13 (15)	Arthroplasty	59	4.5	12
Masada et al 2003	Prospective Cohort	Intramedullary	16 (18)	Arthrodesis	58	2.25	10
Murphy et al 2003	Prospective Comparative Cohort	Dorsal plate, pin, or rod fixation<br>Universal (3)	Arthrodesis – 23 (27)<br>Arthroplasty – 22 (24)	Arthrodesis vs Arthroplasty	51.5	3.1	18

Onuma et al 2015	Retrospective Cohort	Wrist fusion rod	4 (6)	Arthrodesis	56	3.6	14
Pfanner et al 2017	Retrospective Cohort	Universal 2 (4)	22 (23)	Arthroplasty	54.9	6.86	13
Radmer et al 2003	Retrospective Cohort	APH (3)	40 (40)	Arthroplasty	48	4.36	11
Radmer et al 1999	Retrospective Cohort	Uncemented (3)	30 (30)	Arthroplasty	49.3	1.5	12
Rauhaniemi et al 2005	Retrospective Cohort	Intramedullary	115 (115)	Arthrodesis	56	4	14
Rehak et al 2000	Retrospective Cohort	Plate or pin fixation	57 (57)	Arthrodesis	55	2.42	9
Summers et al 1984	Retrospective Cohort	Swanson (1)<br>Meuli (1)	10 (12)	Arthroplasty	54.6	3.4	13
Takwale et al 2002	Retrospective Cohort	Biaxial (3)	63 (66)	Arthroplasty	59.3	4.33	12
Toma et al 2007	Retrospective Cohort	Mannerfeldt or AO plate	37 (42)	Arthrodesis	47.4	6.33	10
Vahvanen et al 1984	Retrospective Cohort	Modified Mannerfeldt	38 (45)	Arthrodesis	50.1	3.9	8
Vicar et al 1986	Retrospective Comparative Cohort	Millender/Nalebuff	Arthrodesis – 24 (33)	Arthrodesis vs Arthroplasty	51.7	5.44	21
		Swanson (1)	Arthroplasty – 28 (37)				
Ward et al 2011	Prospective Cohort	Universal (3)	15 (19)	Arthroplasty	NS	7.3	13

NS – Not stated

Online Table S3. Summary of pain findings included in studies.

Study	Number of patients (number of wrists)	Surgical Intervention	Preoperative pain*	Postoperative pain*	Pain scoring system
Masada et al 2003	16 (18)	Arthrodesis	Present in all patients	No pain reported	Pain present<br>No pain
Onuma et al 2015	4 (6)	Arthrodesis	8	1	0 – 10 point system<br>0 = no pain<br>10 = severe pain
Rauhaniemi et al 2005	115 (115)	Arthrodesis	3.6	1.9	1 – no pain<br>2 – mild pain<br>3 – moderate pain<br>4 – severe pain
Toma et al 2007	37 (42)	Arthrodesis	NS	None: 24<br>Mild: 17<br>Moderate: 0<br>Severe: 1	None<br>Mild<br>Moderate<br>Severe
Vicar et al 1986	24 (33)*	Arthrodesis	None: 0<br>Mild: 3<br>Slight: 14<br>Moderate: 7<br>Severe: 9	None: 22<br>Mild: 10<br>Slight: 1<br>Moderate: 0<br>Severe: 0	None<br>Mild<br>Slight<br>Moderate<br>Severe
	28 (37)	Arthroplasty	None: 0<br>Mild: 3<br>Slight: 9<br>Moderate: 15<br>Severe: 10	None: 25<br>Mild: 8<br>Slight: 4<br>Moderate: 0<br>Severe: 0	

Cobb et al 1996	52 (64)	Arthroplasty	None: 1<br>Mild: 10<br>Moderate: 27<br>Severe: 21<br>Not recorded: 5	None: 46<br>Mild: 9<br>Moderate: 2<br>Severe: 0<br>Not recorded: 7	None<br>Mild<br>Moderate<br>Severe
Figgie, H.E. et al 1988	32 (38)	Arthroplasty	NS	None or slight – 35<br>Moderate – 1<br>Severe – 2	None<br>Mild<br>Moderate<br>Severe
Figgie, M. et al 1990	26 (35)	Arthroplasty	25	48	0 – 50 point system<br>0 = severe pain<br>50 = no pain
Harlingen et al 2014	29 (32)	Arthroplasty	NS	0	0 – 10 point system<br>0 = no pain<br>10 = severe pain
Herzberg et al 2012	129 (129)	Arthroplasty	NS	48 improvement	Visual analogue scale<br>0 – 100 points
Kistler et al 2005	16 (18)	Arthroplasty	6.5	1.8	Visual analogue scale<br>0 = no pain<br>10 = severe pain
Lirette et al 1995	13 (15)	Arthroplasty	NS	50	Hospital for Special Surgery Wrist Scoring System<br>0 = severe pain<br>50 = no pain
Pfanner et al 2017	22 (23)	Arthroplasty	9	0.82	Visual analogue scale<br>0 = no pain<br>10 = severe pain
Radmer et al 2003	40 (40)	Arthroplasty	NS	None: 35<br>Moderate: 5<br>Severe: 0	None<br>Moderate<br>Severe

Radmer et al 1999	30 (30)	Arthroplasty	NS	None: 26<br>Moderate: 4<br>Severe: 0	None<br>Moderate<br>Severe
Summers et al 1984	10 (12)	Arthroplasty	2.75	0.41	0 – 3 point system<br>0 = no pain<br>1 = slight pain<br>2 = moderate pain<br>3 = severe pain
Takwale et al 2002	63 (66)	Arthroplasty	NS	None: 41<br>Mild: 9<br>Moderate: 11<br>Severe: 0<br>Not reported: 2	None<br>Mild<br>Moderate<br>Severe

*numerical values represent the mean for entire patient pool unless otherwise stated.
Stand-alone values represent the mean score in their corresponding pain scale assessment

Online Table S4. Summary of mean range of motion for arthrodesis and arthroplasty.

Study	Flexion/extension		Radial/ulnar deviation		Pronation/supination		Surgical modality
	Pre-op	Post-op	Pre-op	Post-op	Pre-op	Post-op	
Rehak et al 2000	NS	3°	NS	3°	NS	NS	Arthrodesis
Vicar et al 1986	53°	3°	22°	4°	135°	145°	Arthrodesis
Cobb et al 1996	57°	65°	21°	30°	134°	141°	Arthroplasty
Divelbiss et al 2002	48°	55°	17°	98°	138°	168°	Arthroplasty
Ferreres et al 2011	NS	70°	NS	27°	NS	NS	Arthroplasty
Figgie, H.E. et al 1988	NS	38°	NS	20°	NS	NS	Arthroplasty
Figgie, M. et al 1990	35°	50°	NS	20°	NS	NS	Arthroplasty
Harlingen et al 2014	39°	57°	9°	29°	NS	NS	Arthroplasty
Herzberg et al 2012	NS	58°	NS	29°	NS	NS	Arthroplasty
Kistler et al 2005	NS	43°	NS	21°	NS	NS	Arthroplasty
Lirette et al 1995	NS	70°	NS	NS	NS	NS	Arthroplasty
Pfanner et al 2017	NS	72°	NS	25°	NS	NS	Arthroplasty
Radmer et al 2003	NS	69°	NS	24°	NS	NS	Arthroplasty
Radmer et al 1999	37°	68°	21°	NS	122°	172°	Arthroplasty
Summers et al 1984	44°	43°	NS	NS	NS	NS	Arthroplasty
Takwale et al 2002	NS	46°	NS	20°	NS	NS	Arthroplasty
Vicar et al 1986	66°	61°	16°	28°	134°	150°	Arthroplasty
Ward et al 2011	48°	62°	21°	25°	104°	154°	Arthroplasty

^{o} = degree, such that 360° denotes a full rotation

Online Table S5. Summary of patient satisfaction reported for arthrodesis and arthroplasty.

Study	Surgical Intervention	Postoperative satisfaction	Notes
Onuma et al 2015	Arthrodesis	8.2	Not satisfied (0) to very satisfied (10)
Rauhaniemi et al 2005	Arthrodesis	9% worse; 6% unchanged; 43% a little better; 42% much better	Subjective categorization
Rehak et al 2000	Arthrodesis	100% satisfied	Dichotomous categorization
Toma et al 2007	Arthrodesis	21 very satisfied<br>14 satisfied<br>7 unsatisfied	Subjective categorization
Cobb et al 1996	Arthroplasty	Much better: 29<br>Better: 14<br>Same: 2<br>Worse: 2<br>Not recorded: 17	Subjective categorization
Ferreres et al 2011	Arthroplasty	100% satisfied	Dichotomous categorization
Figgie, M. et al 1990	Arthroplasty	Excellent: 20<br>Good: 8<br>Fair: 3<br>Poor: 2<br>Revised: 2	Subjective categorization
Harlingen et al 2014	Arthroplasty	Very satisfied: 18<br>Satisfied: 9<br>Moderately satisfied: 4<br>Not satisfied: 1	Subjective categorization
Herzberg et al 2012	Arthroplasty	88% satisfied or very satisfied	Subjective categorization
Kistler et al 2005	Arthroplasty	Excellent: 5<br>Good: 12<br>Satisfactory: 6<br>Poor: 2	Subjective categorization
Lirette et al 1995	Arthroplasty	100% satisfied	Dichotomous categorization
Radmer et al 1999	Arthroplasty	Satisfied: 26<br>Not satisfied: 4	Subjective categorization
Summers et al 1984	Arthroplasty	Highly satisfied: 8<br>Non-commital: 1<br>Disappointed: 3	Subjective categorization

Online Table S6. Summary of complications.

Study	Surgical intervention	Number of patients (Number of cases)	Description of complications (Number of cases)
Cobb et al 1996	Arthroplasty	52 (64)	Loosening of distal component (8)<br>Infection (1)<br>Dislocation (1)<br>Progressive soft tissue imbalance (1)
Divelbiss et al 2002	Arthroplasty	19 (22)	Palmar dislocation (3)
Ferreres et al 2011	Arthroplasty	14 (14)	Wound dehiscence (1)<br>Post-operative bleeding (1)
Figgie, H.E. et al 1988	Arthroplasty	32 (38)	Re-operation for pain (1)<br>Loosening (1)
Figgie, M. et al 1990	Arthroplasty	26 (35)	Re-operation for pain (1)<br>Loosening (1)
Harlingen et al 2014	Arthroplasty	29 (32)	Dislocation (7)<br>Infection (3)<br>Limited range of motion (7)<br>Nerve damage (3)
Herzberg et al 2012	Arthroplasty	129 (129)	Loosening (4)<br>Wrist ankylosis (1)<br>Deep infection (1)<br>Carpal tunnel syndrome (3)
Kistler et al 2005	Arthroplasty	18 (18)	NS
Lirette et al 1995	Arthroplasty	13 (15)	Dislocation (1)
Masada et al 2003	Arthrodesis	16 (18)	K-wire disengagement (2)<br>Carpal tunnel syndrome (2)
Murphy et al 2003	Comparative	Arthrodesis – 23 (27)	Tendon adhesion (6)<br>Tendon rupture (1)<br>Tenolysis (2)<br>Carpal tunnel syndrome (4)<br>Wound dehiscence (3)<br>CRPS (1)<br>Haematoma (1)<br>Hardware-related fracture (1)
		Arthroplasty – 22 (24)	Wound dehiscence (6)<br>Infection (2)<br>Prosthetic instability (4)<br>Carpal tunnel syndrome (2)
Onuma et al 2015	Arthrodesis	4 (6)	Tendon adhesion (1)<br>Rod fracture (1)<br>Proximal metacarpal fracture (1)

Pfanner et al 2017	Arthroplasty	22 (23)	Implant revision (6)<br>Skin necrosis (1)
Radmer et al 2003	Arthroplasty	40 (40)	Extensor tendon rupture (2)<br>Prosthetic infection (1)
Radmer et al 1999	Arthroplasty	30 (30)	Dislocation (2)
Rauhaniemi et al 2005	Arthrodesis	115 (115)	Radiocarpal joint fusion failure (3)<br>Intraoperative fracture (1)<br>Carpal tunnel syndrome (3)<br>Superficial wound infection (2)
Rehak et al 2000	Arthrodesis	57 (57)	Loss of distal fixation in metacarpal (1)<br>Radial fracture proximal to plate (1)<br>Discomfort (2)<br>Screw loosening (2)
Summers et al 1984	Arthroplasty	10 (12)	Bony ankylosis (1)<br>Prosthesis subluxation through skin and later infection (1)<br>Radiological evidence of loosening (1)
Takwale et al 2002	Arthroplasty	63 (66)	Fractures (4)<br>Dislocations (6)<br>CRPS (1)<br>Impingement (2)<br>Stiffness (4)<br>Ulnar instability (1)
Toma et al 2007	Arthrodesis	37 (42)	Loosening (6)
Vahvanen et al 1984	Arthrodesis	38 (45)	Extensor irritation (4)<br>Pin breakage (1)<br>Median nerve paresis (1)
Vicar et al 1986	Comparative	Arthrodesis – 24 (33)	Carpal tunnel syndrome (3)<br>Distal tenderness (2)<br>Haematoma with delayed wound healing (1)
		Arthroplasty – 28 (37)	Haematoma (2)<br>Prosthesis misplacement (1)<br>Persistent pain (1)<br>Implant fracture (3)<br>Radial carpal impaction (2)
Ward et al 2011	Arthroplasty	15 (19)	Delayed wound healing (4)<br>Instability (1)
